# Identification of prognostic biomarkers and correlations with immune infiltrates among cGAS-STING in hepatocellular carcinoma

**DOI:** 10.1042/BSR20202603

**Published:** 2020-10-16

**Authors:** Zhenhua Qi, Fang Yan, Dongtai Chen, Wei Xing, Qiang Li, Weian Zeng, Bingtian Bi, Jingdun Xie

**Affiliations:** 1 Department of Anesthesiology, Sun Yat-Sen University Cancer Center, State Key Laboratory of Oncology in Southern China, Collaborative Innovation for Cancer Medicine, Guangzhou, Guangdong 510060, China; 2 Department of Clinical Trial Center, Sun Yat-Sen University Cancer Center, State Key Laboratory of Oncology in Southern China, Collaborative Innovation for Cancer Medicine, Guangzhou, Guangdong 510060, China

**Keywords:** cGAS-STING, hepatocellular carcinoma, prognosis, tumor-infiltrating

## Abstract

The cyclic GMP-AMP synthase-stimulator of interferon genes (cGAS-STING) pathway induces innate immunity by activating the production of inflammatory cytokines and type I interferons. Recently, studies revealed that self-DNA from by-products of chromosome instability and tumors could activate the cGAS-STING pathway, and subsequently promote or inhibit tumor development. However, the prognostic value and correlations with immune infiltrates of the cGAS-STING pathway in hepatocellular carcinoma (HCC) have not been clarified. In the present study, we used the Molecular Signatures Database, Oncomine, UALCAN, Human Protein Atlas, Kaplan–Meier plotter, LinkedOmics, and Tumor Immune Estimation Resource databases. Overexpression of XRCC5, IRF3, TRIM21, STAT6, DDX41, TBK1, XRCC6, TREX1, PRKDC, and TMEM173 was markedly correlated with clinical stages and pathological grades in HCC. Moreover, higher mRNA expression of XRCC5, XRCC6, and PRKDC was significantly related with shorter overall survival. However, higher mRNA expression of IFI16, STAT6, NLRC3, and TMEM173 was associated with favorable overall survival. Our results suggested that the kinase targets of the cGAS-STING pathway included the SRC family of tyrosine kinases (LCK and LYN), phosphoinositide 3-kinase-related protein kinase (PIKK) family kinases (ATM and ATR), and mitogen-activated protein kinase 1 (MAPK1). Furthermore, we identified significant correlations among the expression of cGAS-STING pathway and infiltration of B cells, CD4+T cells, CD8+ T cells, macrophages, neutrophils, and dendritic cells in HCC. The expression of the cGAS-STING pathway also exhibited strong relationships with diverse immune marker sets in HCC. These findings suggest that cGAS-STING pathway members may be used as prognostic biomarkers and immunotherapeutic targets HCC patients.

## Introduction

Hepatocellular carcinoma (HCC) is the third leading cause of cancer-related mortality worldwide [1]. The 5-year survival rate of patients with advanced liver cancer is poor due to high tumor recurrence, metastasis, and the lack of early diagnostic biomarkers with high sensitivity and specificity [2]. Owing to poor liver function correlated with cirrhosis and extrahepatic metastasis, most patients with HCC are resistant to common cytotoxic therapies [3]. Although doxorubicin was initially viewed as a first-choice drug for advanced HCC, a controlled trial showed that it was related with a low survival rate [4]. The overall life expectancy of patients with advanced HCC does not exceed 1 year even under treatment with sorafenib or regorafenib [5].Therefore, there is an urgent need to identify novel potential prognostic and therapeutic targets that are related with tumor formation and progression in patients with HCC.

Cyclic GMP-AMP synthase (cGAS) is activated to catalyze the synthesis of cyclic GMP-AMP (cGAMP) on binding to DNA. Moreover, cGAMP acts as a second messenger, which binds to and induces the stimulator of interferon genes (STING) [6,7]. The palmitoylation of STING mediates the recruitment and activation of TANK-binding kinase 1 (TBK1) and interferon regulatory factor 3 (IRF3) to produce cytokines, such as type I interferons (IFNs) [8,9]. STING also acts downstream of several other cytosolic DNA sensors, including DEAD-box helicase 41 (DDX41) and interferon gamma inducible protein 16 (IFI16) in the signaling pathway to produce type I IFNs. Knockdown of DDX41 expression inhibited the ability of myeloid dendritic cells (DCs) to mount cytokine and type I IFN responses to DNA, as well as DNA viruses. Lowering the expression of IFI16 by RNA-mediated interference blocked gene induction and activation of IRF3 [10,11]. The cGAS-STING pathway is an important DNA-sensing machinery in innate immunity and viral defense, and critically involved in tumor development [12,13]. Conditional deletion of TBK1 in lung epithelial cells could inhibit tumorigenesis of lung cancer in a mouse model. Besides promoting tumor growth, TBK1 was also involved in tumor-mediated immunosuppression [14]. Signal transducer and activator of transcription 6 (STAT6) is overexpressed in various human cancers, and is a regulator involved in multiple biological processes of cancers. Studies demonstrated that STAT6 silencing could induce apoptosis and growth inhibition in HCC-derived cells [15].

Recently, studies revealed that the prognosis of patients with tumors was correlated with the expression of cGAS-STING pathway members [16,17]. Cancer immunotherapy is an effective treatment against a number of cancers. Thomsen suggested that modulation of the cGAS-STING pathway could affect the tumor progression of HCC, and potentially be used as a treatment in patients with HCC [18]. Through overexpression and RNA interference, Wang et al. demonstrated that cGAS responded to exogenous dsDNA from the DNA damage response, and subsequently triggered the activation of STING/TBK1-mediated innate immunity in chicken liver cancer [19]. XRCC5 variants play a crucial role in determining susceptibility to HCC [20]. A previous study found that aberrant splicing of IRF3 could result in defects in IFN-mediated antiviral defenses in HCC [21]. Research demonstrated that DNA damage repair by XRCC6 could reverse TLR4-deficiency-worsened liver cancer development by recovering immunity to support autophagy and senescence [22]. An *in vivo* study showed that restored expression of IFI16 could effectively promote tumor regression, which could be partly abrogated by inhibition of the induced inflammasome in HCC [23]. These findings suggest that cGAS-STING pathway members are potential prognostic biomarkers and therapeutic targets and may be associated with immune infiltration in patients with HCC. However, the identification of suitable cGAS-STING pathway members for this purpose remains an important problem that requires urgent attention.

In our study, we searched for cGAS-STING pathway genes present in both the Molecular Signatures Database (MSigDB) and Oncomine database, and in relevant literature [7,13]. We selected 13 key genes of the cGAS-STING pathway members, namely XRCC5, IRF3, TRIM21, IFI16, STAT6, NLRC3, DDX41, TBK1, XRCC6, TREX1, PRKDC, cGAS, and STING (also termed TMEM173). The genes regulated by the cGAS and STING signaling axis include TBK1, IRF3, and STAT6. Moreover, the genes which regulate the pathway include TREX1, DDX41, IFI16, and NLRC3.These signature genes of the cGAS-STING pathway are linked to type I IFN response. First, we compared the expression of cGAS-STING pathway members in HCC tissues and normal tissues via Oncomine, UALCAN, and the Human Protein Atlas. We subsequently analyzed the correlations between these key genes and prognosis in patients with liver cancer using the Kaplan–Meier (K-M) plotter. In addition, we also investigated potential kinase targets of the cGAS-STING pathway members in patients with HCC using LinkedOmics. To further investigate potential immune therapeutic targets, we analyzed the correlations among the expression of cGAS-STING pathway members and immune cell infiltration and diverse immune marker sets in HCC microenvironments obtained from the Tumor Immune Estimation Resource (TIMER) database.

## Materials and methods

### MSigDB analysis

The comprehensive database MSigDB (www.gsea-msigdb.org/gsea/msigdb/) includes >10,000 gene sets and is widely used to perform gene set enrichment analysis [24]. In the present study, we searched this database to obtain cGAS-STING pathway genes.

### Oncomine database analysis

Oncomine (www.oncomine.org) is a cancer microarray database, including 715 datasets and 86,733 samples for DNA or RNA sequence analysis [25]. Oncomine can be used to analyze differences in gene expression between tumor tissues and normal tissues. In the present study, we noted the transcriptional expression of cGAS-STING pathway genes between 20 different cancer samples and their normal adjacent tissues from the Oncomine database. The threshold was determined based on the following values: *P*=0.01, fold change = 1.5, gene rank = 10%, and data type for mRNA.

### UALCAN database analysis

UALCAN (http://ualcan.path.uab.edu) is an interactive web server for the analysis of 31 types of cancer with transcriptome data from The Cancer Genome Atlas (TCGA). This tool is based on clinical data and level 3 RNA-sequence. UALCAN can be used to assess the association of transcriptional expression with relative clinicopathologic features [26]. In our study, UALCAN was utilized to analyze the mRNA expression of cGAS-STING pathway genes in HCC tissues, and estimate their association with clinicopathologic features. A *P*<0.05 denoted statistically significant difference.

### Human protein atlas

The Human Protein Atlas (https://www.proteinatlas.org) is an online site containing immunohistochemistry-based expression data for approximately 20 common types of tumors. It is used to map the human proteins in organs, tissues, and cells through integration of different omics technologies, such as mass spectrometry-based proteomics and transcriptomics [27]. In the present study, the protein expression of different cGAS-STING pathway genes between human normal and HCC tissues was compared using immunohistochemistry images.

### K-M plotter database analysis

The K-M plotter (www.kmplot.com) is used to estimate the effect of 54,000 genes for prognostic analysis in 21 common types of cancer. The database contains gene chip and RNA-sequence data-sources obtained from databases, such as the Gene Expression Omnibus [28,29]. We evaluated the prognostic value of the mRNA expression of cGAS-STING pathway genes in liver cancer via the K-M plotter database. We divided patients according to the median and accepted other default settings prior to the analysis. The hazard ratio (HR) with 95% confidence intervals and log-rank *P*-values was computed. A *P*<0.05 denoted statistically significant differences.

### LinkedOmics

LinkedOmics (http://www.linkedomics.org/) is a publicly available portal containing all 32 types of cancer included in TCGA. It serves as a unique tool for biologists and clinicians to access, compare, and analyze cancer multi-omics data [30]. In this study, we determined the kinase target enrichment of cGAS-STING pathway genes using the “LinkInterpreter” module. We conducted analyses with a number of size of 3 and simulations of 500 in LIHC dataset. We considered 0.05 as the *P*-value cutoff and used the Spearman correlation test to analyze the results statistically.

### TIMER database analysis

TIMER (https://cistrome.shinyapps.io/timer/) is a reliable database for estimating immune cell infiltration using data from TCGA, including 10,897 samples from 32 types of cancer [31]. In the present study, we initially analyzed the expression of cGAS-STING pathway genes in HCC. Subsequently, we examined the correlation between the expression of cGAS-STING pathway members and immune cell infiltrates, including B cells, CD8+ T cells, CD4+ T cells, macrophages, DCs, and neutrophils with gene modules. Highly expressed genes in the tumor microenvironment tend to have negative correlations with tumor purity [32]. The generated scatterplots suggest statistical significance and provide the purity-corrected partial Spearman’s rho value. Further, correlations between the expression of cGAS-STING pathway members and gene markers of immune cells were further evaluated with correlation modules. The cGAS-STING pathway genes were represented on the *X*-axis, and related marker genes were used for the y-axis. Options for partial correlation under the condition of tumor purity were conducted.

### Statistical analysis

The results generated from Oncomine were displayed with fold changes, *P*-values, and *t*-test results. Survival curves were generated via K-M plots. The results of KM plots were presented by *P*-values and HR obtained from a log-rank test. A *P*<0.05 denoted statistically significant differences.

## Results

### mRNA expression levels of cGAS-STING pathway members in HCC

**Figure 1:**
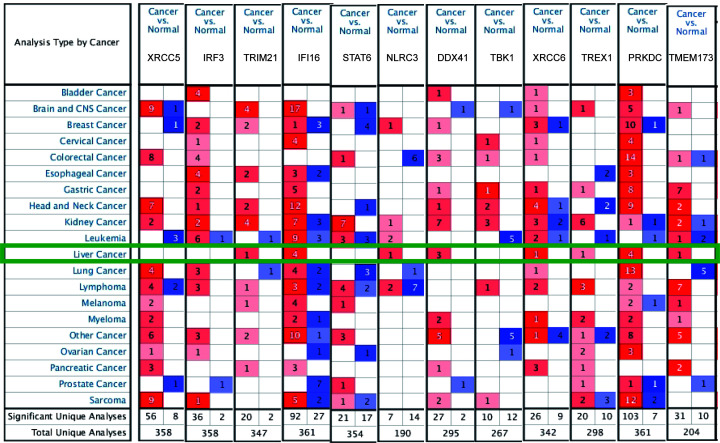
Transcriptional expression of 12 cGAS-STING pathway members in 20 different types of cancer (Oncomine database) Differences in transcriptional expression were compared using Student’s *t*-test. The cutoff criteria were as follows: *P*=0.01, fold change = 1.5, gene rank = 10%, and data type of mRNA.

We analyzed the differences in the transcriptional levels of cGAS-STING pathway members in patients with HCC using the Oncomine database. The results revealed that the mRNA expression of TRIM21, IFI16, NLRC3, DDX41, XRCC6, TREX1, PRKDC, and TMEM173 was significantly higher in HCC tissues in multiple datasets (Figure 1 and Table 1). In the Wurmbach Liver dataset [33], higher expression of TRIM21 was revealed in HCC tissues compared with normal adjacent tissues (fold change = 1.607 and *P* = 4.78E-04). Up-regulation of IFI16 was also found in HCC tissues compared with normal adjacent tissues. The data from the Mas Liver dataset [34] showed increase of 3.474-fold (*P* = 4.23E-14) and 4.715-fold (*P* = 1.91E-16), respectively. Wurmbach [33] observed a 3.649-fold (*P* = 1.02E-06) and 2.250-fold (*P* = 4.06E-04) increase, respectively, in IFI16 mRNA expression in HCC samples. Wurmbach [33] also found a 2.197-fold (*P* = 1.35E-05) increase in NLRC3 mRNA expression in HCC tissues. Similarly, in the Roessler Liver dataset [35], 2.012-fold (*P* = 7.60E-8) and 1.701-fold (*P* = 2.95E-41) increases in DDX41 mRNA expression were found in HCC samples compared with normal tissues. Wurmbach [33] observed a 1.719-fold (*P* = 5.25E-7) increase in DDX41 mRNA expression in HCC samples. In the Roessler Liver 2 dataset [35], high expression of XRCC6 was observed in liver cancer tissues compared with normal adjacent tissues (fold change = 1.828 and *P* = 1.47E-70, respectively). In addition, TREX1 showed higher expression levels in HCC tissues versus normal tissues in Wurmbach Liver (fold change = 1.552 and *P*=0.002) [33]. Similarly, results from three datasets suggested that PRKDC expression was higher in HCC tissue versus normal adjacent tissue [33,35,36]. The mRNA expression of cGAS was not found on Oncomine.

**Table 1:** Significant changes in the transcription levels of cGAS-STING pathway members between HCC and normal liver tissues (Oncomine)

	Types of HCC versus liver	Fold change	*P* value	*t*-test	References
TRIM21	Hepatocellular carcinoma	1.607	4.78E-04	3.847	Wurmbach Liver [34]
IFI16	Hepatocellular carcinoma	3.474	4.23E-14	10.895	Mas Liver [35]
	Hepatocellular carcinoma	4.715	1.91E-16	15.089	Mas Liver [35]
	Hepatocellular carcinoma	3.649	1.02E-06	7.012	Wurmbach Liver [34]
	Hepatocellular carcinoma	2.250	4.06E-04	3.861	Wurmbach Liver [34]
NLRC3	Hepatocellular carcinoma	2.197	1.35E-05	5.560	Wurmbach Liver [34]
DDX41	Hepatocellular carcinoma	2.012	7.60E-8	6.619	Roessler Liver [36]
	Hepatocellular carcinoma	1.719	5.25E-7	6.074	Wurmbach Liver [34]
	Hepatocellular carcinoma	1.701	2.95E-41	15.050	Roessler Liver 2 [36]
XRCC6	Hepatocellular carcinoma	1.828	1.47E-70	21.477	Roessler Liver 2 [36]
TREX1	Hepatocellular carcinoma	1.552	0.002	3.172	Wurmbach Liver [34]
PRKDC	Hepatocellular carcinoma	2.792	4.91E-72	23.447	Roessler Liver 2 [36]
	Hepatocellular carcinoma	3.011	9.67E-9	7.102	Wurmbach Liver [34]
	Hepatocellular carcinoma	1.651	8.50E-15	8.386	Chen Liver [37]
	Hepatocellular carcinoma	2.439	6.57E-8	7.031	Roessler Liver [36]

**Figure 2:**
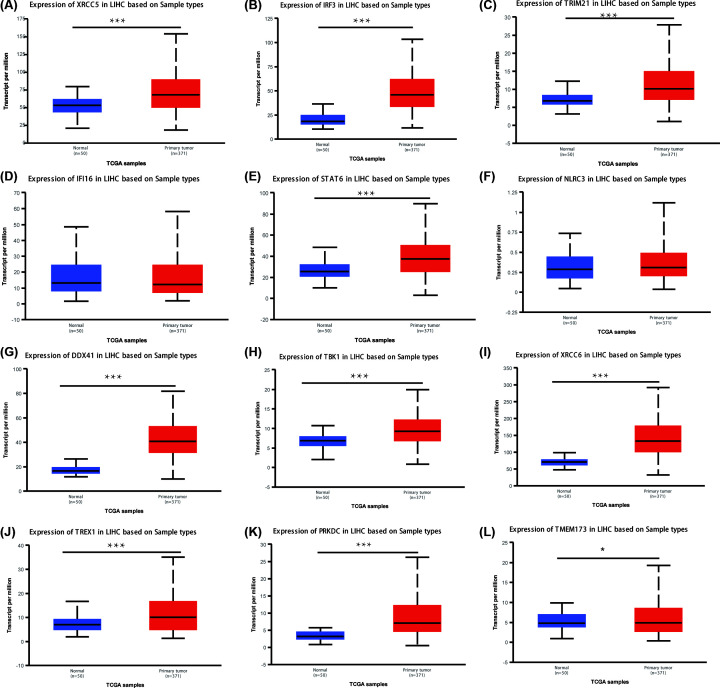
mRNA expression of different cGAS-STING pathway members in HCC samples and adjacent normal liver samples (UALCAN) The mRNA expression of XRCC5, IRF3, TRIM21, STAT6, DDX41, TBK1, XRCC6, TREX1, PRKDC, and TMEM173 was found to be up-regulated in HCC tissues versus normal tissues (**A–C,E,G–L**). The transcriptional levels of IFI16 and NLRC3 did not show significant differences (**D** and **F**); ***P<0.001.

We also investigated the mRNA expression levels of cGAS-STING pathway members in HCC tissue with UALCAN. As shown in Figure 2, the transcriptional levels of XRCC5, IRF3, TRIM21, STAT6, DDX41, TBK1, XRCC6, TREX1, PRKDC (all *P*<0.001) and TMEM173 (*P*<0.05) in HCC tissues were significantly upregulated (Figure 2A–C,E,G–L). However, the transcriptional levels of IFI16 (*P* = 7.04E-02) and NLRC3 (*P* = 9.95E-02) did not show significant differences (Figure 2D,F). The mRNA expression of cGAS was not found on UALCAN.

**Figure 3:**
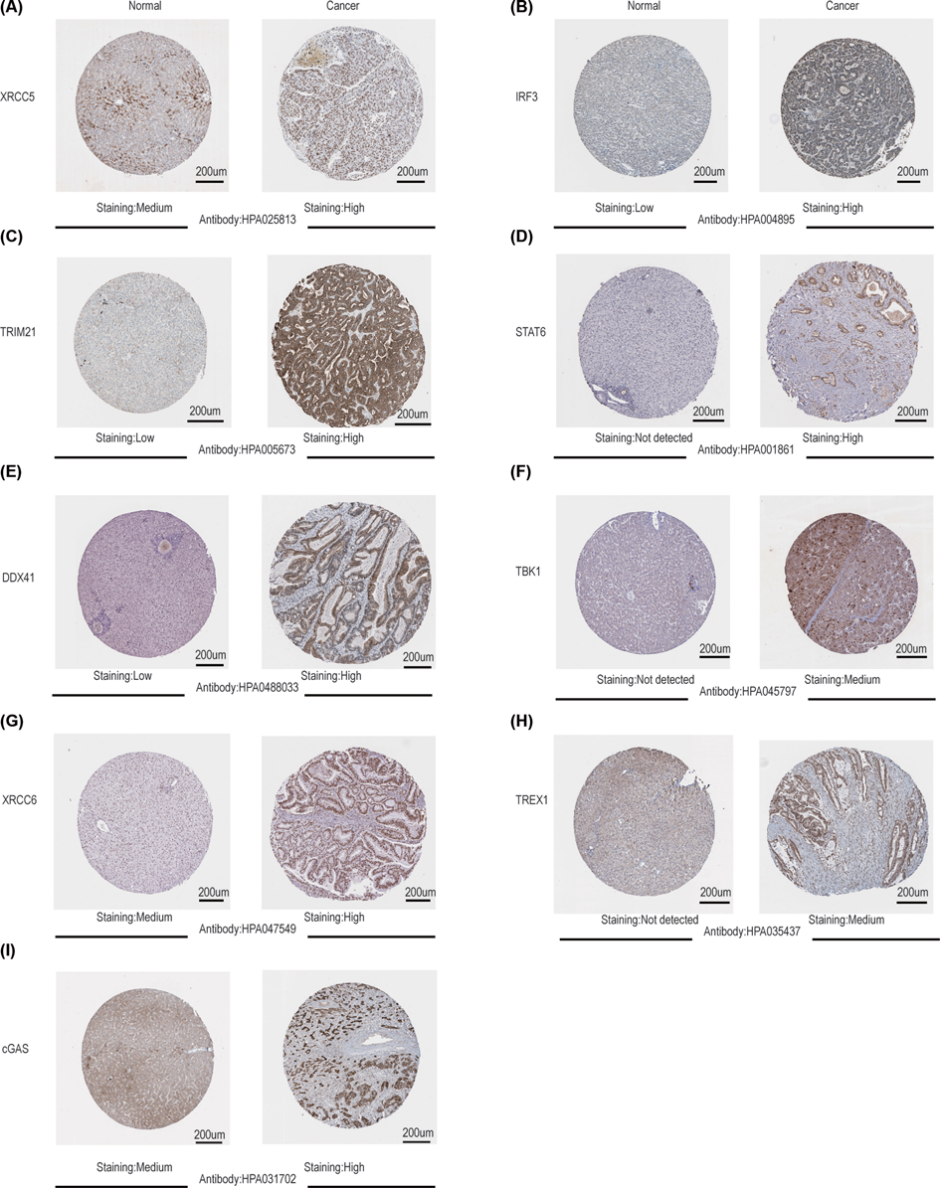
Representative immunohistochemistry images of different cGAS-STING pathway members in HCC tissues and normal liver tissues (Human Protein Atlas) STAT6, TBK1, and TREX1 proteins were not expressed in normal liver tissues, whereas they exhibited medium or high expression in HCC samples (**D,F,H**). In addition, low or medium protein expression of XRCC5, IRF3, TRIM21, DDX41, XRCC6, and cGAS was observed in normal liver tissues; high expression of these proteins was noted in HCC tissues (**A–C,E,G,I**).

After confirming the up-regulation of mRNA expression levels of cGAS-STING pathway members in HCC tissues, we subsequently assessed their protein expression levels based on the Human Protein Atlas website. As shown in Figure 3, STAT6, TBK1, and TREX1 proteins were not expressed in normal liver tissues, whereas they exhibited medium or high expressions in HCC samples (Figure 3D,F,H). In addition, low or medium protein expression of XRCC5, IRF3, TRIM21, DDX41, XRCC6, and cGAS was observed in normal liver tissues, while high protein expression was detected in HCC tissues (Figure 3A–C,E,G,I). However, the immunohistochemical images of IFI16, NLRC3, PRKDC, and TMEM173 expression were not found in this website. In general, these results revealed that the transcription and protein levels of most cGAS-STING pathway members were upregulated in patients with HCC.

**Figure 4:**
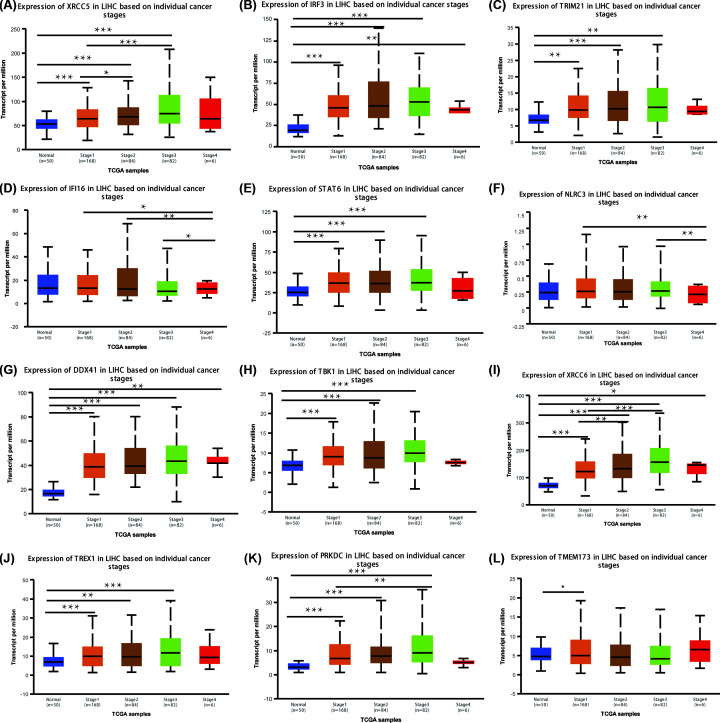
Relationship between the mRNA expression of different cGAS-STING pathway members and individual cancer stages in patients with HCC The mRNA expression of 10 cGAS-STING pathway members was strongly related with the cancer stages of individual patients. Patients who were in more advanced stages tended to express higher mRNA levels of cGAS-STING pathway members. The highest mRNA expression of DDX41 was found in stage 4 (**G**), while the highest mRNA expression of XRCC5, IRF3, TRIM21, STAT6, TBK1, XRCC6, TREX1, and PRKDC was found in stage 3 (**A–C,E,H–K**). The highest mRNA expression of TMEM173 was found in stage 1 (**L**). However, the mRNA expression of IFI16 and NLRC3 did not show a correlation with tumor grades in patients with liver cancer (**D,F**); *P<0.05, **P<0.01, ***P<0.001.

**Figure 5:**
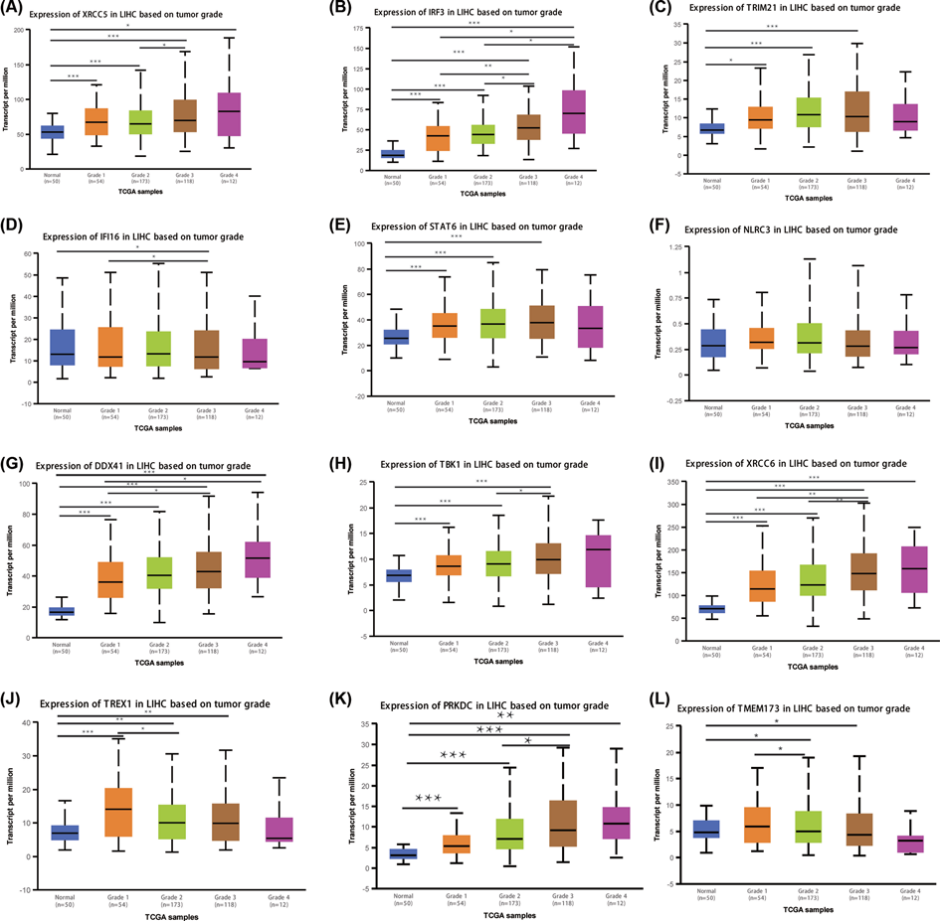
Association of mRNA expression of different cGAS-STING pathway members with tumor grades in patients with HCC The mRNA expression of 10 cGAS-STING pathway members was significantly related with tumor grades; as the tumor grade increased, the mRNA expression of cGAS-STING pathway members tended to increase in parallel. The highest mRNA expression of XRCC5, IRF3, DDX41, TBK1, XRCC6, and PRKDC was found in grade 4 tumors (**A,B,G–I,K**), while the highest mRNA expression of TRIM21, STAT6, and TMEM173 was found in grade 3 tumors (**C,E,L**). However, the highest mRNA expression of IFI16 and TREX1 was found in grade 1 or 2; as the tumor grade increased, the mRNA expression of IFI16 and TREX1 tended to decrease (**D,J**). NLRC3 mRNA expression did not show a correlation with tumor grades in patients with liver cancer (**F**); *P<0.05, **P<0.01, ***P<0.001.

We next analyzed the correlation between the mRNA expression of cGAS-STING pathway members and the pathological stage of patients with liver cancer according to UALCAN. The results are presented in Figures 4 and 5. The mRNA expression of cGAS-STING pathway members, except for IFI16 and NLRC3, was significantly related with individual cancer stages and tumor grades in HCC. Furthermore, patients in advanced stages and tumor grades of HCC had higher mRNA expression levels of cGAS-STING pathway members compared with those in early stages of the disease. In Figure 4, the highest mRNA expression of DDX41 was found in stage 4 (Figure 4G), while the highest mRNA expression of XRCC5, IRF3, TRIM21, STAT6, TBK1, XRCC6, TREX1, and PRKDC was found in stage 3 (Figure 4A–C,E,H–K), which may be attributed to the small sample numbers (only six patients with stage 4 HCC). Figure 4L shows that the highest mRNA expression of TMEM173 was found in stage 1. Similarly, the highest mRNA expression of XRCC5, IRF3, DDX41, TBK1, XRCC6, and PRKDC was found in grade 4 tumors (Figure 5A,B,G–I,K), while the highest mRNA expression of TRIM21, STAT6, and TMEM173 was found in grade 3 (Figure 5C,E,L). However, the highest mRNA expression of IFI16 and TREX1 was found in grade 1 or 2; as the tumor grade increased, their mRNA expression tended to be lower (Figure 5D,J). These results suggested that XRCC5, IRF3, TRIM21, STAT6, DDX41, TBK1, XRCC6, TREX1, PRKDC, and TMEM173 were strongly correlated with tumorigenesis and tumor progression in patients with HCC.

### Prognostic value of mRNA expression of cGAS-STING pathway members among patients with HCC

**Figure 6:**
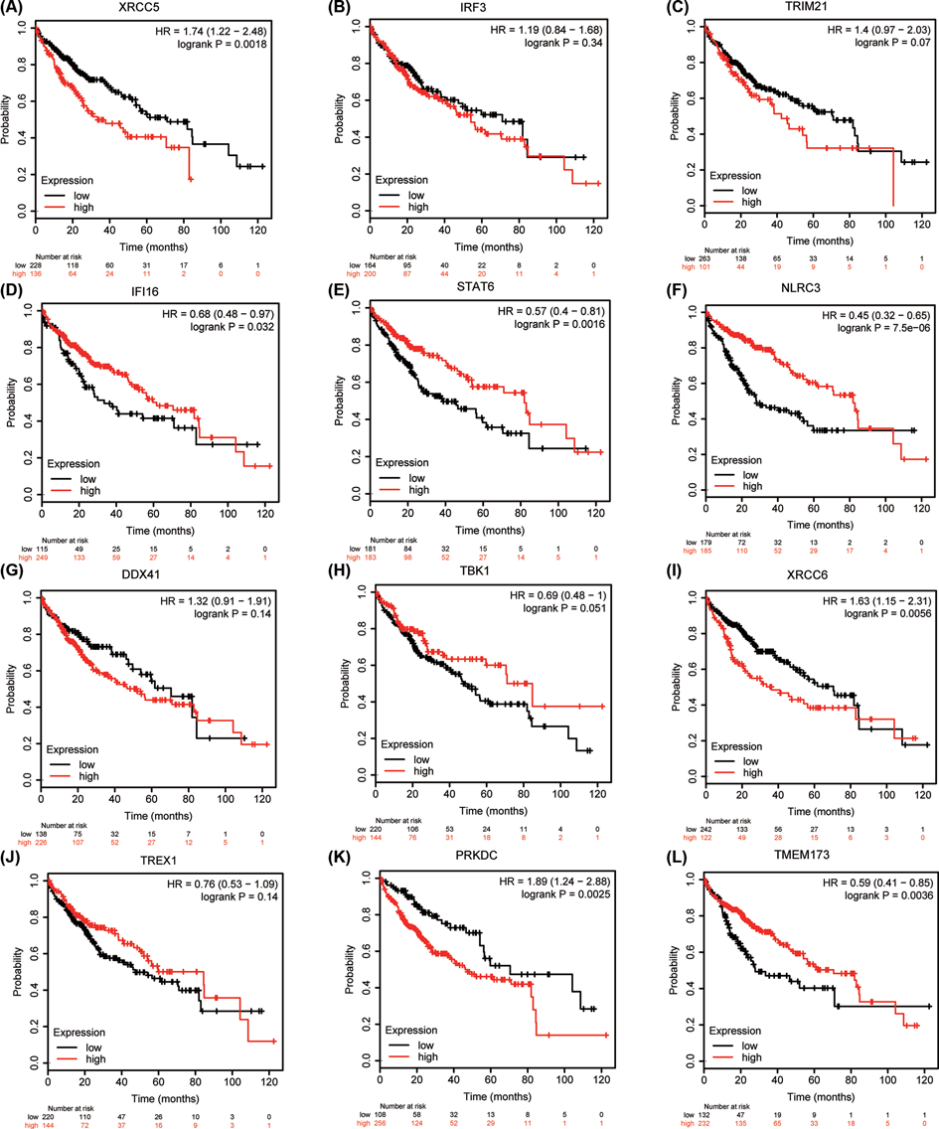
Prognostic value of mRNA expression of different cGAS-STING pathway members in patients with HCC (Kaplan–Meier plotter) Higher mRNA expression of XRCC5, XRCC6, and PRKDC was significantly associated with shorter OS in patients with liver cancer (**A,I,K**). In contrast, higher mRNA expression of IFI16, STAT6, NLRC3, and TMEM173 was significantly related with favorable OS in patients with liver cancer (**D–F,L**). However, IRF3, TRIM21, DDX41, TBK1, and TREX1 mRNA expression did not show a correlation with prognosis in patients with HCC (**B,C,G,H,J**).

We further analyzed the prognostic potential of mRNA expression of cGAS-STING pathway members in patients with HCC using the K-M plotter. Overall survival (OS) curves are illustrated in Figure 6. Higher mRNA expression of XRCC5 (HR: 1.74, 95% confidence interval [CI]: 1.22-2.48, *P*=0.0018), XRCC6 (HR: 1.63, 95% CI: 1.15–2.31, *P*=0.0056), and PRKDC (HR: 1.89, 95% CI: 1.24–2.88, *P*=0.0025) were related with shorter OS (Figure 6A,I,K). In contrast, higher mRNA expression of IFI16 (HR: 0.68, 95% CI: 0.48–0.97, *P*=0.032), STAT6 (HR: 0.57, 95% CI: 0.4–0.81, *P*=0.0016), NLRC3 (HR: 0.45, 95% CI: 0.32–0.65, *P* = 7.5e-06), and TMEM173 (HR: 0.59, 95% CI: 0.41–0.85, *P*=0.0036) was correlated with favorable OS (Figure 6D–F,L). However, the mRNA expression of IRF3 (*P*=0.34), TRIM21 (*P*=0.07), DDX41 (*P*=0.14), TBK1(*P*=0.051), and TREX1 (*P*=0.14) did not show a correlation with prognosis in patients with HCC (Figure 6B,C,G,H,J). These results suggested that the mRNA expression of XRCC5, XRCC6, PRKDC, IFI16, STAT6, NLRC3, and TMEM173 was associated with the prognosis of patients with HCC. The prognostic data of cGAS were not found in the K-M plotter.

### Kinase targets of cGAS-STING pathway members in HCC

Based on the significantly different expression of cGAS-STING pathway members in HCC tissues versus normal tissues, we estimated possible kinase targets of the differentially expressed cGAS-STING pathway members. In the present study, we investigated the top two kinase targets of cGAS-STING pathway members via the LinkedOmics database. As shown in Table 2, PLK1 and ATR were the top two kinase targets of XRCC5. DYRK1A and EGFR were noted as the targets for the IRF3 kinase-target network. LYN and LCK were mainly correlated with TRIM21. Constituents of the IFI16 kinase-target network were primarily correlated with SYK and LCK. ATR and PLK1, and LCK and SYK were the top two kinase targets of STAT6 and NLRC3, respectively. Mitogen-activated protein kinase 1 (MAPK1) and DYRK1A were regarded as the kinase targets of DDX41. ATM and DAPK1 were mainly related with TBK1. AURKB and PLK1 were shown as kinase targets of XRCC6. ATM and CDK2, as well as ATM and CDK1, were the top two targets of TREX1 and PRKDC, respectively. BUB1 and HCK were regarded as the kinase targets of TMEM173.The kinase targets of cGAS were not found in the LinkedOmics database.

**Table 2:** The kinase target networks of cGAS-STING pathway members in HCC (LinkedOmics)

cGAS-STING pathway	Enriched kinase target	Description	Leading EdgeNum	P value
XRCC5	Kinase_PLK1	Polo-like kinase 1	51	0
	Kinase_ATR	ATR serine/threonine kinase	32	0
IRF3	Kinase_DYRK1A	Dual specificity tyrosine phosphorylation regulated kinase 1A	7	0.004
	Kinase_EGFR	Epidermal growth factor receptor	17	0.008
TRIM21	Kinase_LYN	LYN proto-oncogene, Src family tyrosine kinase	22	0
	Kinase_LCK	LCK proto-oncogene, Src family tyrosine kinase	31	0
IFI16	Kinase_SYK	Spleen associated tyrosine kinase	21	0
	Kinase_LCK	LCK proto-oncogene, Src family tyrosine kinase	25	0
STAT6	Kinase_ATR	ATR serine/threonine kinase	31	0
	Kinase_PLK1	Polo-like kinase 1	32	0
NLRC3	Kinase_LCK	LCK proto-oncogene, Src family tyrosine kinase	24	0
	Kinase_SYK	Spleen associated tyrosine kinase	20	0
DDX41	Kinase_MAPK1	Mitogen-activated protein kinase 1	60	0
	Kinase_DYRK1A	Dual specificity tyrosine phosphorylation regulated kinase 1A	6	0.011
TBK1	Kinase_ATM	ATM serine/threonine kinase	56	0
	Kinase_DAPK1	Death-associated protein kinase 1	6	0
XRCC6	Kinase_AURKB	Aurora kinase B	41	0
	Kinase_PLK1	Polo-like kinase 1	34	0
TREX1	Kinase_ATM	ATM serine/threonine kinase	69	0
	Kinase_CDK2	Cyclin-dependent kinase 2	124	0
PRKDC	Kinase_ATM	ATM serine/threonine kinase	63	0
	Kinase_CDK1	Cyclin-dependent kinase 1	125	0
TMEM173	Kinase_BUB1	BUB1 mitotic checkpoint serine/threonine kinase	4	0.029
	Kinase_HCK	HCK proto-oncogene, Src family tyrosine kinase	10	0.028

### Immune cell infiltration of cGAS-STING pathway members in HCC

**Figure 7:**
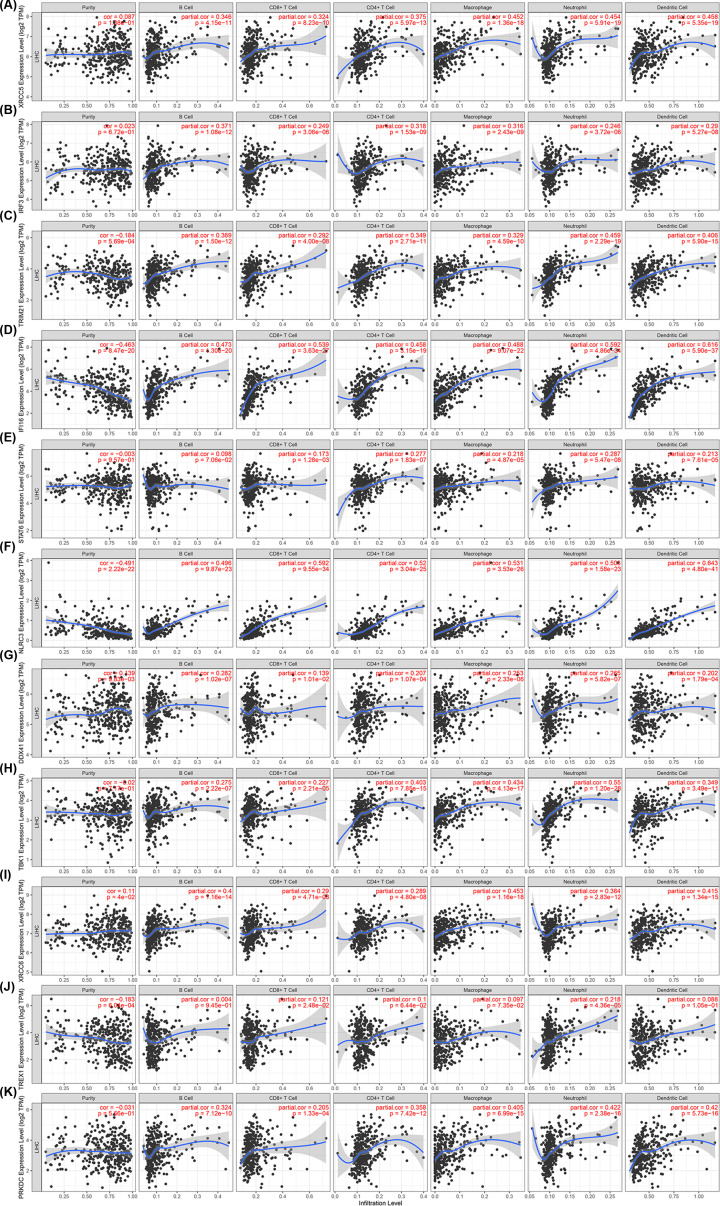
Correlation between differentially expressed cGAS-STING pathway members and immune cell infiltration (TIMER) Relationship between the abundance of immune cells and expression of (**A**) XRCC5, (**B**) IRF3, (**C**) TRIM21, (**D**) IFI16, (**E**) STAT6, (**F**) NLRC3, (**G**) DDX41, (**H**) TBK1, (**I**) XRCC6, (**J**) TREX1, and (**K**) PRKDC in HCC.

Tumor-infiltrating lymphocytes can be used as independent predictors for sentinel lymph node status and survival in cancer [37]. Therefore, we investigated the correlations between differentially expressed cGAS-STING pathway members and the infiltration of immune cells using the TIMER database. As shown in Figure 7, there was a positive relationship between the expression of XRCC5, IRF3, TRIM21, IFI16, NLRC3, DDX41, TBK1, XRCC6, and PRKDC, and the infiltration of B cells, CD4^+^ T cells, CD8^+^ T cells, neutrophils, macrophages, and DCs (Figure 7A–D,F–I,K). STAT6 expression (Figure 7E) was positively associated with the infiltration of CD8^+^ T cells (correlation: 0.173, *P* = 1.28e−03), macrophages (correlation: 0.218, *P* = 4.87e−05), neutrophils (correlation: 0.287, *P* = 5.47e−08), CD4^+^ T cells (correlation: 0.277, *P* = 1.83e−07), and DCs (correlation: 0.213, *P* = 7.61e−05). Similarly, the expression of TREX1 (Figure 7J) was correlated with the infiltration of CD8+ T cells (correlation: 0.121, *P* = 2.48e−02), and neutrophils (correlation: 0.218, *P* = 4.36e−05). These results strongly suggested that, in patients with liver cancer, the cGAS-STING pathway may act a specific role for immune cell infiltration, including B cells, CD8^+^ T cells, CD4^+^ T cells, macrophages, neutrophils, and DCs.

### Correlation analysis between the expression of the cGAS-STING pathway and immune marker sets

We investigated the relationships between cGAS-STING pathway members and different marker genes of immune cells of HCC using the TIMER database. The immune marker sets of various immune cells, such as monocytes, M1 and M2 macrophages, tumor-associated macrophages (TAMs), and DCs were analyzed. In addition, we investigated various functional T cells, such as T helper 1 (Th1), Th2, and regulatory T (Treg) cells. The results are presented in Supplementary Tables S1–3. After correlation adjustment by purity, we found that the expression levels of cGAS-STING pathway members were significantly related with most immune marker sets of different immune cells and various functional T cells in liver cancer.

Importantly, the expression of immune marker sets of TAMs, monocytes, M1 and M2 macrophages was associated with the expression levels of most cGAS-STING pathway members, including XRCC5, TRIM21, IFI16, STAT6, NLRC3, TBK1, XRCC6, and PRKDC (Supplementary Tables S1–3).

Furthermore, the results also suggested that the expression of most marker sets of DCs, such as HLA-DPB1, CD1C, NRP1, and ITGAX had strong correlations with XRCC5, TRIM21, IFI16, STAT6, NLRC3, TBK1, XRCC6, and PRKDC expression in HCC (Supplementary Tables S1–3). These results suggested that there was a strong correlation between cGAS-STING pathway members and DC infiltration. In addition, there was a significant connection between FOXP3 and TGFβ1 for Treg cells and cGAS-STING pathway members in HCC.

Significant correlations between cGAS-STING pathway members, (e.g., XRCC5, TRIM21, IFI16, STAT6, NLRC3, TBK1, XRCC6, and PRKDC) and marker gene sets of Treg and T cell exhaustion (e.g., FOXP3, STAT5B, CCR8, PDCD1, TGFβ1, LAG3, CTLA4, and HAVCR2) are also presented in Supplementary Tables S1–3. The cGAS-STING pathway members exhibited significant correlations with immune infiltrating cells in HCC, and may play an important role in immune escape in the liver cancer microenvironment.

## Discussion

The cGAS-STING pathway has emerged as a potential mechanism to induce inflammation-mediated tumorigenesis. Actually, persistent activation of this pathway and its downstream effectors, such as TBK1, has been connected with chronic inflammation and cancer progression [38,39]. The development of HCC is a multistep process that involves continuous inflammatory damage, such as hepatocyte necrosis [40]. Some studies have observed correlations between cGAS-STING pathway members, the tumor microenvironment, and cancer immunotherapy. However, the prognostic value and potential therapeutic targets of the cGAS-STING pathway in HCC are poorly characterized. In the present study, we investigated the expression, prognostic values, and correlations with immune infiltrates of different cGAS-STING pathway members in HCC.

We first investigated the expression of cGAS-STING pathway members and its correlations with the pathological stage in HCC. Based on the Oncomine database, the mRNA expression of TRIM21, IFI16, NLRC3, DDX41, XRCC6, TREX1, PRKDC, and TMEM173 was significantly higher in HCC tissues in multiple datasets. Moreover, in patients with HCC, high expression of mRNA and protein was found in cGAS-STING pathway member genes, including XRCC5, IRF3, TRIM21, STAT6, DDX41, TBK1, XRCC6, TREX1, PRKDC, and TMEM173. Furthermore, the mRNA expression of these 10 genes was strongly associated with cancer stages and tumor grades in patients with HCC. These data demonstrate that differentially expressed cGAS-STING pathway members may play a significant role in HCC. Recently, high expression of XRCC5 has been found in breast and gastric cancer [17,41]. A strong correlation was found between the variable number tandem repeat polymorphism in the XRCC5 gene and the risk of breast cancer [17]. Overexpression of XRCC5 was also detected in gastric cancer, in which XRCC5 regulated the overexpression of chloride channel 3 (CLC3) [41]. Additionally, Liu [42] found that higher expression of XRCC5 was related with metastasis through the Wnt/β-catenin signaling pathway in patients with HCC, which was consistent with our results. In conclusion, XRCC5 may participate in the tumorigenesis of HCC.

Recently, Shi found that pharmacological targeting or knockdown of IRF3 using amlexanox. This drug is used for anti-inflammatory treatment, and can inhibit gastric tumor growth in a Yes-associated protein-dependent manner. The expression of IRF3 is up-regulated and prognosticates patient survival in gastric cancer [43]. A study showed that the expression of TBK1 was increased in mesenchymal small cell lung cancer cell lines [44]. Research found that TRIM21 modulated epithelial–mesenchymal transition (EMT) by regulating the stability of Snail in breast cancer [45]. To our knowledge, this was the first study to investigate the correlation among IRF3, TBK1, and TRIM21 with HCC. Our results suggested that all three may participate in the tumorigenesis of HCC.

The potential prognostic value of the mRNA expression levels of cGAS-STING pathway members in patients with HCC was subsequently investigated. The findings revealed that higher mRNA expression of XRCC5, XRCC6, and PRKDC was significantly associated with shorter OS. In contrast, higher mRNA expression of IFI16, STAT6, and NLRC3 was significantly related with better OS. Liu [42] observed that the high mRNA expression of XRCC5 predicted poor prognosis in patients with HCC, which was in accordance with our results. Studies have shown that NLRC3 was a negative regulator in innate immune signaling activated by STING. NLRC3 could inhibit STING–TBK1 interaction and the production of downstream type I IFN [46]. IFI16 enhanced STING activation by interacting with STING via the PYRIN domain. Moreover, the IFI16-induced inflammasome was found to inhibit HCC growth and metastasis, and was a tumor suppressor during the development [23]. In our study, we found that NLRC3 and IFI16 were independent prognostic factors for favorable OS in patients with liver cancer.

We also investigated the kinase targets of the cGAS-STING pathway members. We discovered that the SRC family of tyrosine kinases (LCK and LYN), phosphoinositide 3-kinase-related protein kinase (PIKK) family kinases (ATM and ATR), and MAPK1 were potential kinase targets of the cGAS-STING pathway members. These kinase targets affect the progression of cell cycle, DNA damage, and EMT [47–51]. Furthermore, they are involved in tumor progression by mediating tumor cell invasion, apoptosis, and migration [51–53]. As a result, differentially expressed cGAS-STING pathway members may modulate DNA repair, the EMT, and cell cycle progression by regulating these kinases in patients with HCC.

Another important finding in the present study is that the expression of cGAS-STING pathway members is associated with various immune cell infiltration levels in liver cancer. The cGAS-STING pathway can mediate protective immune defense toward infection with a great number of DNA-containing pathogens, as well as generate intrinsic antitumor immunity [54]. The accumulation of tumor DNA could activate STING-IRF3-induced IFN signaling to enforce tumor-antigen presentation on DCs and cross-prime CD8^+^ T cells for antitumor immunity [55]. There is an increasing body of evidence supporting that immune cell infiltration could affect cancer recurrence and progression, and also play an important role in clinical outcome and response to immunotherapy [56,57]. CD4^+^ T cells may be involved in the recognition of tumor antigens, and the activation of M1 macrophages may mediate the inhibition of tumor growth [58]. Our results demonstrate that there is a significant correlation between the expression of cGAS-STING pathway members and the infiltration of immune cells, such as T cells, macrophages, and DCs. This implies that cGAS-STING pathway members may act as potential prognostic indicators, as well as reflect the immune status. STING activation in hepatic macrophages could induce the production of proinflammatory cytokines, leading to nonalcoholic steatohepatitis that is characterized by hepatic steatosis [59].

Moreover, the correlations between the expression of cGAS-STING pathway members and the marker sets of immune cells suggest the role of cGAS-STING pathway members in regulating tumor immunology in HCC. Firstly, the M1 macrophage markers (e.g., IRF5 and PTGS2) and the marker genes of M2 macrophages (e.g., VSIG4, and MS4A4A) were significantly associated with the expression of XRCC5, TRIM21, IFI16, STAT6, NLRC3, TBK1, XRCC6, and PRKDC. Collectively, the results suggest the regulatory role of these cGAS-STING pathway genes in the polarization of TAMs. Moreover, our results reveal a strong relationship between the expression of XRCC5, TRIM21, IFI16, STAT6, NLRC3, TBK1, XRCC6, and PRKDC and DC infiltration. These genes also play a potential role in activating Treg cells and inducing T-cell exhaustion. DCs promote tumor metastasis by reducing the cytotoxicity of CD8^+^ T cells and increasing Treg cells [60]. Further studies are warranted to investigate whether these genes are important factors in inducing the DCs and tumor metastasis.

In addition, the increase in the expression of XRCC5, TRIM21, IFI16, STAT6, NLRC3, TBK1, XRCC6, and PRKDC was significantly related with the expression of Treg and T-cell exhaustion gene markers, such as FOXP3, STAT5B, TGFβ1, PDCD1, CTLA4, HAVCR2, and LAG3 in patients with HCC. HAVCR2 is an important surface protein from exhausted T cells [61]. In human squamous cell carcinomas, STING signaling abrogated tumor immunogenicity by recruiting Treg cells [62]. This is highly correlated with the expression of XRCC5, TRIM21, IFI16, STAT6, NLRC3, TBK1, XRCC6, and PRKDC in HCC. Furthermore, significant associations were also found between the expression of XRCC5, TRIM21, IFI16, STAT6, NLRC3, TBK1, XRCC6, and PRKDC and the regulation of some marker genes of T helper cells (e.g., Th1, Th2, and Th17). These associations suggest the potential mechanism through which these cGAS-STING pathway members may regulate T-cell functions in HCC. Collectively, the results suggest that cGAS-STING pathway members (e.g., XRCC5, TRIM21, IFI16, STAT6, NLRC3, TBK1, XRCC6, and PRKDC) may play a crucial role in the regulation and recruitment of immune infiltrating cells in HCC.

The present study had some limitations. First, all the data in the present study were obtained from online databases; hence, further studies involving larger sample sizes, and *in vitro* and *in vivo* experiments are warranted to confirm our results. Second, analysis based on the transcriptional levels may indicate a few aspects of the immune status; however, its ability to detect global changes is limited. Finally, we did not analyze the potential mechanisms of cGAS-STING pathway members in HCC. Future studies should be performed to investigate the detailed mechanism among cGAS-STING pathway members and HCC.

## Conclusions

XRCC5, IRF3, TRIM21, STAT6, DDX41, TBK1, XRCC6, TREX1, PRKDC, and TMEM173 were found to be significantly positive correlated with clinical cancer stages and tumor grades in patients with liver cancer. In addition, high mRNA expression of XRCC5, XRCC6, and PRKDC was significantly related with poor OS. In contrast, high mRNA expression of IFI16, STAT6, NLRC3, and TMEM173 was strongly correlated with favorable OS in HCC. Increased expression levels of cGAS-STING pathway members are correlated with increased infiltration levels of immune cells, including B cells, CD8^+^ T cells, CD4^+^ T cells, neutrophils, macrophages, and DCs in HCC. Therefore, cGAS-STING pathway members, especially XRCC5, IFI16, STAT6, NLRC3, XRCC6, and PRKDC, are likely involved in immune infiltration and can be used as potential prognostic biomarkers for patients with HCC. We hope our results provide a new perspective for the design of new immunotherapeutic drugs against HCC.

## Supplementary Material

Supplementary Tables S1-S3

## Data Availability

The data that support the findings of our study are openly available from Molecular Signatures Database (MSigDB), Oncomine database, UALCAN, Human Protein Atlas, Kaplan-Meier plotter, LinkedOmics, and Tumor Immune Estimation Resource (TIMER) database (https://www.gsea-msigdb.org/gsea/msigdb/, www.oncomine.org, http://ualcan.path.uab.edu, https://www.proteinatlas.org, www.kmplot.com, http://www.linkedomics.org/, https://cistrome.shinyapps.io/timer/).

## Abbreviations

cGAMP: cyclic GMP-AMP
cGAS: cyclic GMP-AMP synthase
DC: dendritic cell
DDX41: DEAD-box helicase 41
HCC: hepatocellular carcinoma
IFI16: interferon gamma inducible protein 16
IRF3: interferon regulatory factor 3
MAPK1: mitogen-activated protein kinase 1
PIKK: phosphoinositide 3-kinase-related protein kinase
STAT6: signal transducer and activator of transcription-6
STING: stimulator of interferon response cGAMP interactor
TBK1: TANK-binding kinase 1
TMEM173: transmembrane protein 173
