# Supplementary material for: Identification of prognostic biomarkers and correlations with immune infiltrates among cGAS-STING in hepatocellular carcinoma
Source: Biosci Rep. 2020 Oct 16;40(10):BSR20202603. doi: 10.1042/BSR20202603 (PMC7569205; doi:10.1042/BSR20202603)
Supplement: Supplementary Tables S1-S3 [file BSR-2020-2603_supp.pdf]

Supplementary Table 1 Correlation analysis among XRCC5, IRF3, TRIM21, and IFI16 and related genes and markers of immune cells in TIMER.

Description	Gene Markers	XRCC5				IRF3				TRIM21				IFI16			
		None		Purity		None		Purity		None		Purity		None		Purity	
		Cor	P	Cor	P	Cor	P	Cor	P	Cor	P	Cor	P	Cor	P	Cor	P
CD8+ T cell	CD8A	0.179	***	0.253	***	0.202	***	0.231	***	0.330	***	0.279	***	0.579	***	0.458	***
	CD8B	0.118	*	0.185	***	0.290	***	0.321	***	0.256	***	0.204	***	0.475	***	0.340	***
T cell (general)	CD3D	0.111	*	0.178	***	0.413	***	0.458	***	0.350	***	0.328	***	0.515	***	0.388	***
	CD3E	0.146	**	0.241	***	0.267	***	0.329	***	0.372	***	0.334	***	0.642	***	0.517	***
	CD2	0.131	*	0.220	***	0.320	***	0.377	***	0.388	***	0.358	***	0.610	***	0.485	***
B cell	CD19	0.165	**	0.192	***	0.307	***	0.325	***	0.270	***	0.213	***	0.437	***	0.328	***
	CD79A	0.066	0.205	0.130	*	0.266	***	0.293	***	0.241	***	0.161	**	0.535	***	0.402	***
Monocyte	CD86	0.322	***	0.430	***	0.254	***	0.301	***	0.451	***	0.426	***	0.692	***	0.588	***
TAM	CSF1R	0.277	***	0.381	***	0.181	***	0.217	***	0.439	***	0.403	***	0.682	***	0.568	***
	CCL2	0.186	***	0.255	***	0.109	*	0.126	*	0.383	***	0.341	***	0.635	***	0.523	***
	CD68	0.246	***	0.309	***	0.157	**	0.172	**	0.328	***	0.281	***	0.484	***	0.349	***
	IL10	0.264	***	0.339	***	0.127	*	0.151	**	0.313	***	0.248	***	0.551	***	0.425	***
M1 Macrophage	NOS2	0.150	**	0.155	***	−0.132	*	−0.122	*	0.151	**	0.134	*	0.241	***	0.232	***
	IRF5	0.393	***	0.382	***	0.325	***	0.324	***	0.416	***	0.436	***	0.247	***	0.275	***
	PTGS2	0.317	***	0.419	***	0.087	0.096	0.106	0.050	0.312	***	0.255	***	0.622	***	0.503	***

M2 Macrophage	CD163	0.253	***	0.334	***	−0.03	0.562	−0.022	0.683	0.409	***	0.362	***	0.557	***	0.426	***
	VSIG4	0.230	***	0.308	***	0.069	0.188	0.094	0.081	0.433	***	0.399	***	0.576	***	0.450	***
	MS4A4A	0.230	***	0.325	***	0.028	0.594	0.051	0.349	0.424	***	0.401	***	0.633	***	0.516	***
Neutrophils	CEACAM8	0.084	0.106	0.095	0.079	0.033	0.523	0.051	0.341	−0.016	0.756	−0.027	0.620	0.108	*	0.078	0.149
	ITGAM	0.273	***	0.319	***	0.248	***	0.273	***	0.467	***	0.460	***	0.569	***	0.492	***
	CCR7	0.134	**	0.215	***	0.143	**	0.180	***	0.312	***	0.247	***	0.593	***	0.451	***
Natural killer cell	KIR2DL1	0.052	0.317	0.026	0.627	−0.068	0.189	−0.094	0.081	0.008	0.875	−0.034	0.534	0.144	**	0.118	*
	KIR2DL3	0.160	**	0.188	***	0.126	*	0.140	**	0.252	***	0.214	***	0.290	***	0.246	***
	KIR2DL4	0.157	**	0.175	**	0.194	***	0.207	***	0.315	***	0.291	***	0.376	***	0.334	***
	KIR3DL1	0.132	*	0.146	**	−0.048	0.356	−0.074	0.170	0.117	*	0.074	0.170	0.207	***	0.180	***
	KIR3DL2	0.121	*	0.157	**	0.128	*	0.141	**	0.126	*	0.086	0.110	0.280	***	0.204	***
	KIR3DL3	0.075	0.148	0.076	0.159	0.012	0.821	−0.01	0.852	0.024	0.646	−0.006	0.906	0.121	*	0.085	0.113
	KIR2DS4	0.176	***	0.185	***	0.018	0.734	0.031	0.566	0.051	0.326	0.061	0.257	0.166	**	0.195	***
Dendritic cell	HLA-DPB1	0.232	***	0.309	***	0.218	***	0.249	***	0.447	***	0.416	***	0.709	***	0.614	***
	HLA-DQB1	0.153	**	0.219	***	0.242	***	0.264	***	0.364	***	0.334	***	0.576	***	0.456	***
	HLA-DRA	0.266	***	0.346	***	0.148	**	0.170	**	0.513	***	0.489	***	0.708	***	0.613	***
	HLA-DPA1	0.275	***	0.361	***	0.130	*	0.159	**	0.487	***	0.466	***	0.719	***	0.629	***
	CD1C	0.258	***	0.320	***	0.158	**	0.170	**	0.282	***	0.240	***	0.523	***	0.416	***
	NRP1	0.532	***	0.552	***	0.053	0.312	0.049	0.362	0.243	***	0.221	***	0.457	***	0.433	***
	ITGAX	0.294	***	0.377	***	0.284	***	0.333	***	0.421	***	0.398	***	0.619	***	0.518	***
Th1	TBX21	0.141	**	0.208	***	0.137	**	0.170	**	0.302	***	0.240	***	0.496	***	0.366	***

	STAT4	0.228	***	0.270	***	0.256	***	0.248	***	0.395	***	0.372	***	0.506	***	0.451	***
	STAT1	0.488	***	0.526	***	0.277	***	0.279	***	0.576	***	0.552	***	0.561	***	0.535	***
	IFNG	0.140	**	0.191	***	0.268	***	0.297	***	0.303	***	0.276	***	0.399	***	0.302	***
	TNF	0.258	***	0.346	***	0.271	***	0.315	***	0.422	***	0.391	***	0.543	***	0.439	***
Th2	GATA3	0.223	***	0.326	***	0.190	***	0.225	***	0.349	***	0.298	***	0.627	***	0.532	***
	STAT6	0.380	***	0.365	***	0.024	0.645	0.012	0.821	0.312	***	0.289	***	0.239	***	0.261	***
	STAT5A	0.352	***	0.389	***	0.309	***	0.315	***	0.324	***	0.295	***	0.526	***	0.454	***
	IL13	0.074	0.153	0.062	0.252	0.156	**	0.150	**	0.168	**	0.144	**	0.134	**	0.113	*
Tfh	BCL6	0.361	***	0.353	***	0.046	0.380	0.066	0.220	0.142	**	0.152	**	0.194	***	0.246	***
	IL21	0.078	0.132	0.116	*	0.099	0.056	0.125	*	0.150	**	0.142	**	0.168	**	0.136	*
Th17	STAT3	0.321	***	0.344	***	−0.016	0.759	−0.01	0.856	0.443	***	0.420	***	0.519	***	0.487	***
	IL17A	0.094	0.070	0.108	*	−0.005	0.921	0.014	0.793	0.042	0.421	0.048	0.374	0.137	**	0.146	**
Treg	FOXP3	0.242	***	0.274	***	−0.046	0.375	−0.016	0.773	0.378	***	0.362	***	0.330	***	0.270	***
	CCR8	0.386	***	0.458	***	0.134	**	0.147	**	0.428	***	0.400	***	0.579	***	0.519	***
	STAT5B	0.500	***	0.490	***	−0.063	0.227	−0.063	0.244	0.251	***	0.296	***	0.128	*	0.243	***
	TGFB1	0.319	***	0.398	***	0.362	***	0.392	***	0.284	***	0.225	***	0.539	***	0.422	***
T cell exhaustion	PDCD1	0.183	***	0.245	***	0.402	***	0.442	***	0.300	***	0.252	***	0.497	***	0.380	***
	CTLA4	0.155	**	0.218	***	0.410	***	0.454	***	0.299	***	0.267	***	0.496	***	0.375	***
	LAG3	0.149	**	0.177	***	0.355	***	0.361	***	0.272	***	0.238	***	0.303	***	0.212	***
	HAVCR2	0.303	***	0.411	***	0.283	***	0.338	***	0.437	***	0.409	***	0.703	***	0.606	***
	GZMB	0.107	*	0.130	*	0.184	***	0.215	***	0.132	*	0.052	0.334	0.417	***	0.305	***

Supplementary Table 2 Correlation analysis among STAT6, NLRC3, DDX41, and TBK1 and related genes and markers of immune cells in TIMER.

Description	Gene Markers	STAT6				NLRC3				DDX41				TBK1			
		None		Purity		None		Purity		None		Purity		None		Purity	
		Cor	P	Cor	P	Cor	P	Cor	P	Cor	P	Cor	P	Cor	P	Cor	P
CD8+ T cell	CD8A	0.131	*	0.151	**	0.737	***	0.675	***	0.045	0.388	0.100	0.064	0.197	***	0.211	***
	CD8B	0.002	0.966	0.012	0.829	0.612	***	0.528	***	0.027	0.609	0.074	0.172	0.068	0.191	0.075	0.162
T cell (general)	CD3D	−0.006	0.906	0.004	0.946	0.627	***	0.539	***	0.032	0.538	0.093	0.085	0.096	0.066	0.107	*
	CD3E	0.103	*	0.126	*	0.797	***	0.732	***	0.002	0.971	0.067	0.214	0.166	**	0.188	***
	CD2	0.085	0.101	0.111	*	0.783	***	0.721	***	−0.004	0.943	0.057	0.293	0.154	**	0.179	***
B cell	CD19	0.093	0.074	0.077	0.151	0.528	***	0.427	***	0.072	0.164	0.092	0.088	0.174	***	0.162	**
	CD79A	0.025	0.626	0.023	0.670	0.642	***	0.526	***	−0.039	0.450	0.003	0.953	0.071	0.175	0.064	0.235
Monocyte	CD86	0.163	**	0.190	***	0.726	***	0.637	***	0.146	**	0.227	***	0.350	***	0.410	***
	CSF1R	0.205	***	0.236	***	0.699	***	0.595	***	0.137	**	0.216	***	0.303	***	0.358	***
TAM	CCL2	0.165	**	0.188	***	0.617	***	0.487	***	0.047	0.371	0.129	*	0.268	***	0.314	***
	CD68	0.146	**	0.156	**	0.494	***	0.358	***	0.079	0.127	0.128	*	0.289	***	0.314	***
	IL10	0.135	**	0.138	*	0.584	***	0.451	***	0.078	0.136	0.122	*	0.311	***	0.340	***
M1 Macrophage	NOS2	0.216	***	0.216	***	0.235	***	0.216	***	0.093	0.075	0.104	0.054	0.243	***	0.244	***
	IRF5	0.366	***	0.362	***	0.355	***	0.395	***	0.417	***	0.410	***	0.463	***	0.469	***
	PTGS2	0.199	***	0.233	***	0.623	***	0.506	***	0.003	0.949	0.083	0.125	0.367	***	0.437	***
M2 Macrophage	CD163	0.204	***	0.225	***	0.627	***	0.512	***	0.053	0.307	0.097	0.071	0.359	***	0.412	***

	VSIG4	0.194	***	0.231	***	0.565	***	0.436	***	0.111	*	0.176	**	0.322	***	0.385	***
	MS4A4A	0.199	***	0.238	***	0.627	***	0.502	***	0.060	0.251	0.133	*	0.307	***	0.363	***
Neutrophils	CEACAM8	−0.045	0.388	−0.047	0.379	0.061	0.243	0.028	0.606	−0.029	0.580	−0.019	0.720	0.105	*	0.117	*
	ITGAM	0.259	***	0.284	***	0.529	***	0.448	***	0.297	***	0.356	***	0.418	***	0.476	***
	CCR7	0.120	*	0.121	*	0.725	***	0.622	***	−0.085	0.104	−0.04	0.459	0.184	***	0.198	***
Natural killer cell	KIR2DL1	0.074	0.153	0.044	0.418	0.183	***	0.153	**	−0.04	0.442	−0.068	0.210	0.053	0.312	0.031	0.570
	KIR2DL3	0.182	***	0.177	***	0.364	***	0.333	***	0.144	**	0.187	***	0.176	***	0.174	**
	KIR2DL4	0.127	*	0.132	*	0.373	***	0.328	***	0.129	*	0.148	**	0.171	***	0.175	**
	KIR3DL1	0.222	***	0.225	***	0.317	***	0.311	***	−0.013	0.807	***	0.998	0.146	**	0.157	**
	KIR3DL2	0.064	0.222	0.082	0.126	0.389	***	0.347	***	0.070	0.182	0.089	0.099	0.088	0.089	0.101	0.062
	KIR3DL3	0.083	0.109	0.043	0.426	0.167	**	0.137	*	0.059	0.257	0.031	0.561	0.040	0.444	−0.01	0.853
	KIR2DS4	0.210	***	0.218	***	0.276	***	0.319	***	0.063	0.225	0.057	0.292	0.104	*	0.109	*
Dendritic cell	HLA-DPB1	0.163	**	0.183	***	0.728	***	0.640	***	0.124	*	0.184	***	0.205	***	0.229	***
	HLA-DQB1	0.056	0.279	0.066	0.219	0.592	***	0.482	***	0.103	*	0.160	**	0.109	*	0.125	*
	HLA-DRA	0.213	***	0.236	***	0.729	***	0.640	***	0.118	*	0.182	***	0.301	***	0.339	***
	HLA-DPA1	0.206	***	0.225	***	0.748	***	0.667	***	0.111	*	0.179	***	0.299	***	0.340	***
	CD1C	0.144	**	0.153	**	0.658	***	0.580	***	−0.002	0.970	0.054	0.315	0.208	***	0.226	***
	NRP1	0.458	***	−0.003	0.957	0.517	***	0.506	***	0.135	**	0.165	**	0.534	***	0.546	***
	ITGAX	0.218	***	0.245	***	0.683	***	0.588	***	0.172	***	0.235	***	0.392	***	0.442	***
Th1	TBX21	0.160	**	0.174	**	0.727	***	0.655	***	−0.068	0.194	−0.03	0.572	0.166	**	0.175	**
	STAT4	0.159	**	0.173	**	0.565	***	0.522	***	0.012	0.816	0.044	0.411	0.242	***	0.260	***
	STAT1	0.326	***	0.324	***	0.600	***	0.587	***	0.194	***	0.210	***	0.514	***	0.518	***
	IFNG	0.029	0.583	0.044	0.410	0.514	***	0.450	***	0.126	*	0.170	**	0.171	***	0.191	***
	TNF	0.163	**	0.183	***	0.666	***	0.577	***	0.091	*	0.150	**	0.316	***	0.349	***

Th2	GATA3	0.142	**	0.178	***	0.769	***	0.704	***	0.036	0.491	0.093	0.083	0.256	***	0.304	***
	STAT6	1.000	***	−1	***	0.326	***	0.360	***	0.143	**	0.120	*	0.542	***	0.518	***
	STAT5A	0.330	***	0.346	***	0.573	***	0.531	***	0.202	***	0.220	***	0.394	***	0.411	***
	IL13	−0.054	0.303	−0.084	0.118	0.179	***	0.163	**	0.106	*	0.077	0.155	0.108	*	0.105	0.052
Tfh	BCL6	0.374	***	0.368	***	0.199	***	0.239	***	0.118	*	0.111	*	0.427	***	0.431	***
	IL21	0.046	0.378	0.065	0.232	0.235	***	0.212	***	0.013	0.801	0.037	0.495	0.140	**	0.154	**
Th17	STAT3	0.339	***	0.330	***	0.423	***	0.354	***	0.095	0.068	0.101	0.060	0.556	***	0.571	***
	IL17A	0.159	**	0.163	**	0.156	**	0.163	**	−0.067	0.195	−0.05	0.351	0.122	*	0.115	*
Treg	FOXP3	0.168	**	0.155	**	0.470	***	0.425	***	0.083	0.110	0.135	*	0.358	***	0.370	***
	CCR8	0.276	***	0.294	***	0.663	***	0.612	***	0.144	**	0.181	***	0.512	***	0.551	***
	STAT5B	0.496	***	0.486	***	0.309	***	0.456	***	0.244	***	0.217	***	0.648	***	0.660	***
	TGFB1	0.140	**	0.152	**	0.563	***	0.455	***	0.176	***	0.253	***	0.250	***	0.274	***
T cell exhaustion	PDCD1	0.068	0.194	0.063	0.240	0.607	***	0.518	***	0.108	*	0.171	**	0.155	**	0.146	**
	CTLA4	0.034	0.514	0.045	0.407	0.587	***	0.495	***	0.084	0.108	0.147	**	0.147	**	0.161	**
	LAG3	−0.004	0.931	−0.004	0.935	0.456	***	0.407	***	0.033	0.530	0.046	0.389	0.083	0.109	0.079	0.141
	HAVCR2	0.140	**	0.169	**	0.699	***	0.601	***	0.151	**	0.228	***	0.333	***	0.397	***
	GZMB	0.079	0.129	0.058	0.282	0.497	***	0.394	***	0.021	0.689	0.066	0.222	0.069	0.184	0.048	0.374

Supplementary Table 3 Correlation analysis among XRCC6, TREX1, and PRKDC, and related genes and markers of immune cells in TIMER.

Description	Gene Markers	XRCC6				TREX1				PRKDC			
		None		Purity		None		Purity		None		Purity	
		Cor	P	Cor	P	Cor	P	Cor	P	Cor	P	Cor	P
CD8+ T cell	CD8A	0.132	*	0.216	***	0.154	**	0.076	0.157	0.166	**	0.184	***
	CD8B	0.128	*	0.216	***	0.157	**	0.085	0.113	0.083	0.109	0.097	0.071
T cell (general)	CD3D	0.153	**	0.243	***	0.116	*	0.036	0.507	0.139	**	0.159	**
	CD3E	0.122	*	0.235	***	0.112	*	0.006	0.915	0.183	***	0.215	***
	CD2	0.124	*	0.233	***	0.123	*	0.027	0.617	0.172	***	0.208	***
B cell	CD19	0.205	***	0.257	***	0.113	*	0.035	0.512	0.205	***	0.199	***
	CD79A	0.098	0.060	0.199	***	0.067	0.201	−0.041	0.453	0.117	*	0.127	*
Monocyte	CD86	0.322	***	0.454	***	0.177	***	0.100	0.063	0.357	***	0.417	***
	CSF1R	0.265	***	0.388	***	0.227	***	0.150	**	0.301	***	0.353	***
TAM	CCL2	0.125	*	0.199	***	0.253	***	0.195	***	0.182	***	0.189	***
	CD68	0.269	***	0.357	***	0.101	0.052	0.019	0.725	0.344	***	0.378	***
	IL10	0.211	***	0.299	***	0.171	***	0.092	0.088	0.289	***	0.313	***
M1 Macrophage	NOS2	0.035	0.500	0.044	0.420	0.141	**	0.125	0.020	0.165	**	0.165	**
	IRF5	0.371	***	0.375	***	0.142	**	0.130	0.015	0.433	***	0.417	***
	PTGS2	0.161	**	0.247	***	0.160	**	0.093	0.086	0.284	***	0.317	***
M2 Macrophage	CD163	0.181	***	0.271	***	0.167	**	0.093	0.083	0.290	***	0.337	***
	VSIG4	0.173	***	0.268	***	0.185	***	0.120	0.026	0.244	***	0.280	***
	MS4A4A	0.196	***	0.307	***	0.170	**	0.099	0.065	0.271	***	0.324	***
Neutrophils	CEACAM8	0.083	0.112	0.095	0.078	−0.073	0.161	−0.087	0.108	0.066	0.205	0.055	0.312
	ITGAM	0.282	***	0.345	***	0.222	***	0.185	***	0.370	***	0.406	***
	CCR7	0.072	0.166	0.156	**	0.087	0.095	−0.022	0.679	0.181	***	0.201	***
Natural killer cell	KIR2DL1	−0.058	0.266	−0.087	0.108	0.055	0.289	0.015	0.780	0.074	0.155	0.052	0.335
	KIR2DL3	0.070	0.176	0.117	*	0.202	***	0.151	**	0.091	0.080	0.109	*
	KIR2DL4	0.124	*	0.156	**	0.169	**	0.129	*	0.113	*	0.116	*
	KIR3DL1	0.003	0.956	0.034	0.524	0.079	0.129	0.049	0.369	0.092	0.076	0.096	0.075

	KIR3DL2	0.056	0.279	0.098	0.068	0.063	0.226	0.027	0.614	0.111	*	0.127	*
	KIR3DL3	0.042	0.422	0.032	0.551	0.051	0.326	0.027	0.623	0.044	0.403	0.014	0.795
	KIR2DS4	0.053	0.307	0.050	0.350	0.115	*	0.095	0.079	0.088	0.090	0.086	0.111
Dendritic cell	HLA-DPB1	0.226	***	0.323	***	0.205	***	0.117	*	0.222	***	0.242	***
	HLA-DQB1	0.138	**	0.211	***	0.133	*	0.044	0.415	0.157	**	0.162	**
	HLA-DRA	0.230	***	0.329	***	0.210	***	0.131	*	0.290	***	0.327	***
	HLA-DPA1	0.211	***	0.305	***	0.176	***	0.088	0.104	0.272	***	0.306	***
	CD1C	0.181	***	0.236	***	0.029	0.583	−0.058	0.282	0.240	***	0.239	***
	NRP1	0.421	***	0.433	***	0.100	0.055	0.062	0.253	0.500	***	0.506	***
	ITGAX	0.270	***	0.373	***	0.131	*	0.049	0.360	0.409	***	0.458	***
Th1	TBX21	0.041	0.428	0.119	*	0.128	*	0.028	0.608	0.162	**	0.184	***
	STAT4	0.098	0.059	0.138	*	0.083	0.111	0.042	0.438	0.197	***	0.209	***
	STAT1	0.384	***	0.433	***	0.137	**	0.090	0.094	0.450	***	0.468	***
	IFNG	0.148	**	0.222	***	0.139	**	0.084	0.122	0.142	**	0.167	**
	TNF	0.221	***	0.329	***	0.144	**	0.052	0.334	0.329	***	0.382	***
Th2	GATA3	0.128	*	0.235	***	0.122	*	0.023	0.674	0.232	***	0.285	***
	STAT6	0.257	***	0.247	***	0.159	**	0.132	*	0.390	***	0.374	***
	STAT5A	0.350	***	0.392	***	0.262	***	0.230	***	0.369	***	0.385	***
	IL13	−0.035	0.506	−0.039	0.467	0.166	**	0.149	**	0.014	0.789	0.005	0.928
Tfh	BCL6	0.289	***	0.278	***	0.014	0.781	0.017	0.749	0.340	***	0.332	***
	IL21	0.073	0.163	0.104	0.053	0.150	**	0.126	*	0.081	0.118	0.096	0.075
Th17	STAT3	0.214	***	0.243	***	0.094	0.070	0.051	0.348	0.417	***	0.431	***
	IL17A	0.039	0.452	0.042	0.442	−0.08	0.122	−0.069	0.204	0.108	*	0.109	*
Treg	FOXP3	0.084	0.105	0.119	*	0.199	***	0.171	**	0.258	***	0.276	***
	CCR8	0.271	***	0.353	***	0.060	0.250	−0.008	0.886	0.488	***	0.543	***
	STAT5B	0.356	***	0.337	***	0.029	0.582	0.048	0.373	0.533	***	0.540	***
	TGFB1	0.325	***	0.426	***	0.066	0.204	−0.025	0.646	0.306	***	0.338	***
T cell exhaustion	PDCD1	0.226	***	0.318	***	0.129	*	0.038	0.479	0.201	***	0.209	***
	CTLA4	0.212	***	0.294	***	0.063	0.228	−0.013	0.808	0.191	***	0.214	***
	LAG3	0.139	**	0.181	***	0.157	**	0.114	*	0.102	*	0.101	0.062
	HAVCR2	0.291	***	0.424	***	0.173	***	0.100	0.065	0.344	***	0.407	***
	GZMB	0.065	0.211	0.104	0.053	0.188	***	0.117	*	0.054	0.296	0.044	0.415
